# Design and synthesis of novel bis-annulated caged polycycles via ring-closing metathesis: pushpakenediol

**DOI:** 10.3762/bjoc.10.280

**Published:** 2014-11-13

**Authors:** Sambasivarao Kotha, Mirtunjay Kumar Dipak

**Affiliations:** 1 Department of Chemistry, Indian Institute of Technology-Bombay, Powai, India, Fax: 022-2572 7152

**Keywords:** Diels–Alder cycloaddition, Grignard addition, pentacycloundecane (PCUD), ring-closing metathesis

## Abstract

Intricate caged molecular frameworks are assembled by an atom economical process via a Diels–Alder (DA) reaction, a Claisen rearrangement, a ring-closing metathesis (RCM) and an alkenyl Grignard addition. The introduction of olefinic moieties in the pentacycloundecane (PCUD) framework at appropriate positions followed by RCM led to the formation of novel heptacyclic cage systems.

## Introduction

Caged polycyclic compounds draw the attention of synthetic organic chemists due to their unusual reactivity patterns as well as their strained nature [1–9]. Several pentacycloundecane (PCUD) related molecules were found to be key structural elements in various drugs [10–12], high-energy materials [13–15] and supramolecules [16–17].

**Figure 1:**
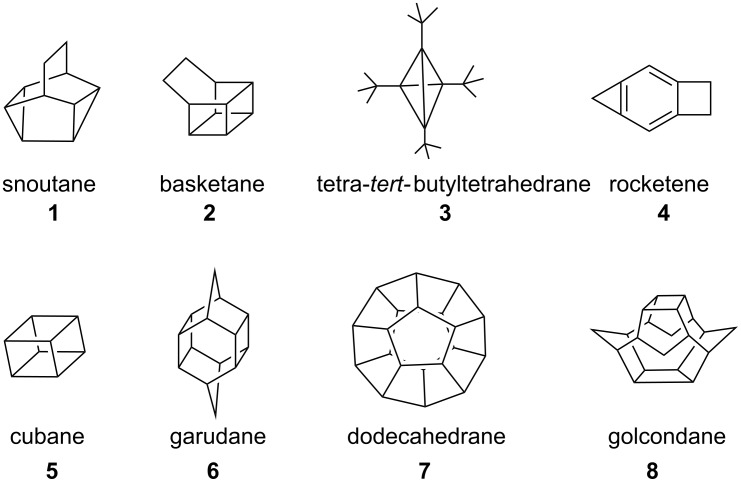
Selected theoretically interesting molecules.

In addition, caged molecules possess unusual and often unique properties that are associated with their rigid carbocyclic framework [1–2]. They are useful synthons in the design and synthesis of natural as well as non-natural products [3–4]. Several intricate targets (Figure 1), such as snoutane (**1**) [18], basketane (**2**) [19], tetrahedrane (**3**) [20], triprismane [21], rocketene (**4**) [22], cubane (**5**) [23], garudane (**6**) [24], dodecahedrane (**7**) [25–26] and golcondane (**8**) [27] were assembled by employing various novel synthetic routes.

## Results and Discussion

**Figure 2:**
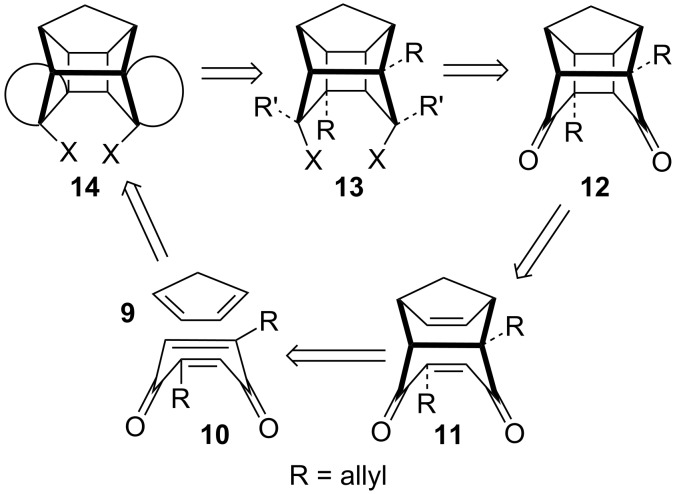
Retrosynthetic approach toward bis-annulated PCUD.

In connection with our interest to prepare annulated PCUD, we proposed various dialkylated pentacyclic diones such as 1,9-dialkylpentacyclo[5.4.0.0^2,6^.0^3,10^.0^5,9^]undeca-8,11-dione **12** by using [4 + 2] and [2 + 2] cycloaddition strategies, which involve the DA reaction of 2,5-dialkyl-1,4-benzoquinone **10** and 1,3-cyclopentadiene (**9**) followed by the formation of the cyclobutane ring through a [2 + 2] photocycloaddition reaction (Figure 2). Later on, one can introduce two allyl groups by using traditional carbonyl chemistry. The addition of alkyl groups can take place from the less hindered *exo* side of this caged system. If the R and R’ groups contain unsaturated systems one can think of constructing additional rings on the pentacyclic framework by utilizing ring-closing metathesis (RCM). By varying the length of unsaturated component one can generate diverse PCUD ring systems.

**Scheme 1:**
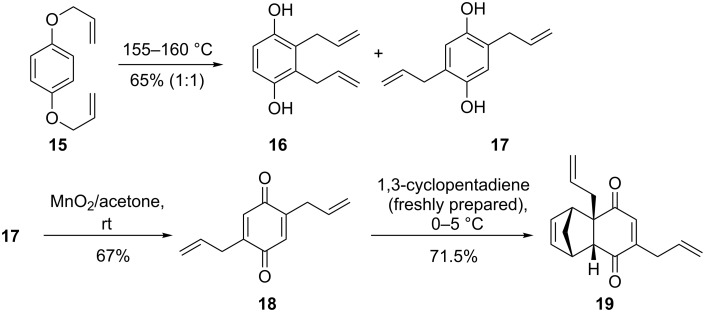
The synthesis of diallylated tricyclic diene **19**.

To realize the strategy depicted in Figure 2, we chose 2,5-diallyl-1,4-benzoquinone (**18**) as a viable option. We deliberately choose two unsaturated R groups at non-vicinal positions, because we have shown in an earlier report that the vicinal allyl groups in PCUD can be converted to a six-membered ring by RCM [28]. Since the two allyl groups in the PCUD framework are far apart, they did not undergo a RCM reaction. Later, by introducing additional unsaturated fragments in the PCUD system one can generate multiple rings in the PCUD system by using the RCM protocol. To this end, we began with the Claisen rearrangement of bis(allyloxy)benzene **15** to deliver the two possible rearranged diallylated products **16** and **17** [29–30] in equimolar ratio. When 2,5-diallyl-1,4-hydroquinone (**17**) was subjected to MnO_2_ oxidation in acetone at room temperature the corresponding 2,5-diallyl-1,4-benzoquinone (**18**) was obtained in good yield (67%). Since the quinone **18** is prone to polymerization (it gave a long streak on TLC when exposed to air at rt), it was immediately subjected to the [4 + 2] cycloaddition reaction with freshly prepared 1,3-cyclopentadiene (**9**) at 0–5 ºC to deliver compound **19** in 71.5% yield (Scheme 1).

The formation of cycloadduct **19** was confirmed on the basis of ^1^H and ^13^C NMR spectral data. In ^1^H NMR data the integration of the olefinic region (ring olefinic proton) corresponds to the three protons and, as the cycloadduct is unsymmetrical, all the seventeen carbons appeared in the ^13^C NMR spectrum.

**Scheme 2:**
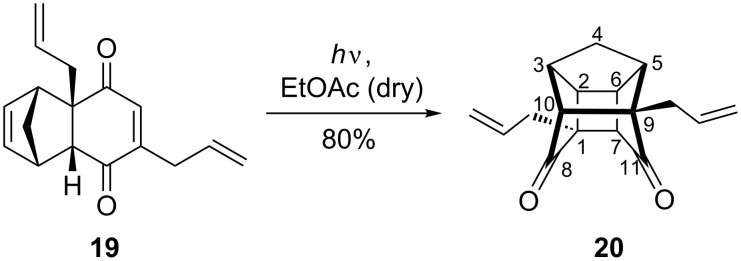
The synthesis of diallylated pentacyclic dione **20**.

The stereochemistry of adduct **19** was expected to be *endo* as the reaction was performed at low temperature [28], and generally, the kinetically controlled product was produced under these reaction conditions. Our assumption was found to be correct, when we found that the DA adduct undergoes a smooth [2 + 2] photocyclization [31] upon exposure to UV light to generate caged dione **20** in good yield (80%). The structure of the photoadduct was assigned as 1,9-diallyl PCUD **20** on the basis of the spectral data (Scheme 2).

**Scheme 3:**
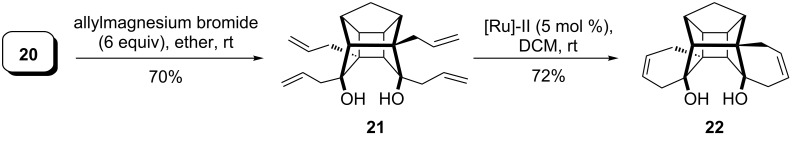
The synthesis of heptacyclic diol **22**.

Having prepared the pentacyclic diallyldione **20**, we ventured into the synthesis of PCUD based novel heptacyclic systems. The dione **20** was subjected to an allyl Grignard addition reaction, which resulted in the formation of tetra-allyldiol **21**. The structure of diol **21** was established on the basis of high field ^1^H NMR (400 MHz) spectral data and further supported by ^13^C NMR spectral data (Scheme 3).

The Grignard addition at a trigonal carbon may result in the formation of C–C bond in two possible ways (*en* face and *zu* face), but due to steric reasons only the *exo*–*exo* allyl addition product was isolated in the present case. The assumption of *exo*–*exo* stereochemistry has been further revived when the tetra-allyldiol **21** underwent a smooth RCM [32–37] upon its exposure to Grubbs’ second generation catalyst to deliver bis-annulated heptacyclic diol **22** in good yield (72%) (Scheme 3).

**Figure 3:**
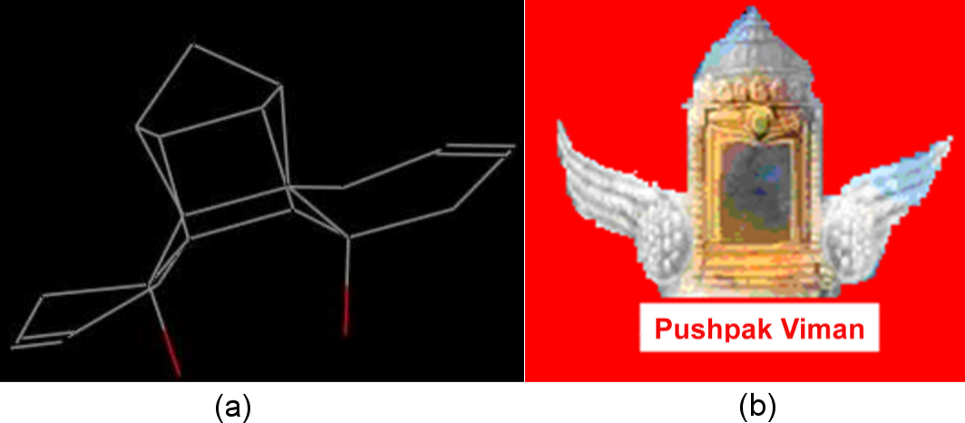
(a) Optimized structure of **22** (b) Ancient flying machine “Pushpak Viman”.

The molecular model and the optimized structure of heptacyclic diol **22** resembles the ancient flying machine “Puspak Viman” designed by the famous ancient aeronautical engineer saint Bhardwaj [38–40]. Therefore, we coined the name ‘pushpakenediol’ [41] for the heptacyclic diol. The optimization of the structure was carried out by means of Chem Draw 3D and the structure was visualized with the Mercury software (Figure 3).

**Scheme 4:**
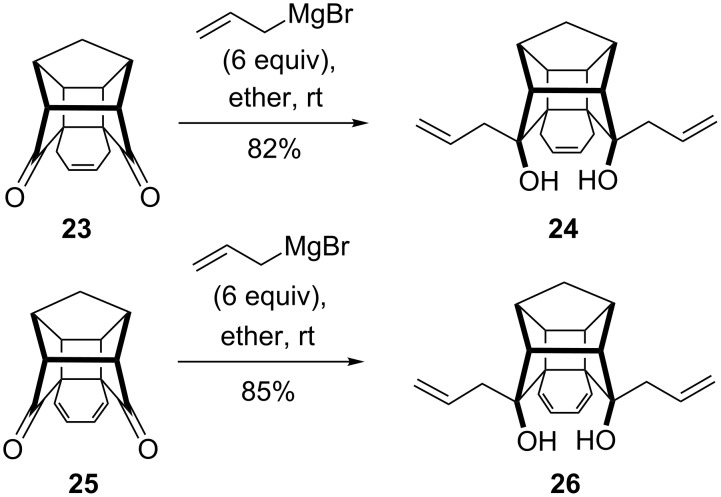
The synthesis of diallylated hexacyclic diols.

We turned our attention toward the next target, i.e., the symmetrical heptacyclic diol **27**, by adopting the ring-rearrangement metathesis (RRM) protocol [42]. To this end, hexacyclic diones **23** [28] and **25** [28] were treated with an excess amount of allylmagnesium bromide (6 equiv) at room temperature to give the desired diallylated adducts **24** and **26** as sole products in 82% and 85% yield, respectively (Scheme 4). The structures of the adducts **24** and **26** were established based on high field ^1^H NMR (400 MHz) spectral data and further supported by ^13^C NMR spectral data.

**Scheme 5:**
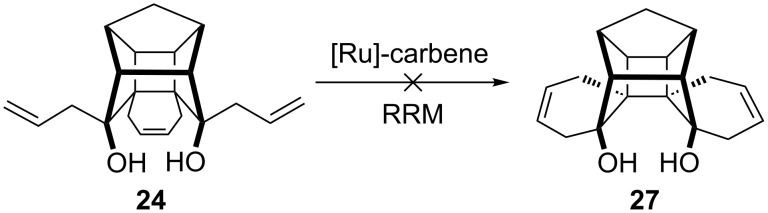
The attempted synthesis of heptacyclic diol via ring-rearrangement metathesis.

It was anticipated that the two allyl groups and the cyclohexene moiety present in **24** would undergo a ring-rearrangement metathesis (RRM), [42] which involves the ring-opening and ring-closing metathesis sequence (ROM–RCM) in a single step to generate the novel heptacyclic system **27**. However, even under harsh reaction conditions, we did not observe the formation of the required RRM product (Scheme 5).

The RRM reaction was carried out under various reaction conditions with different metathesis catalysts, for example, with Grubbs’ 1^st^ and 2^nd^ generation catalysts at rt as well as under reflux conditions. However, even the presence of ethylene during the metathesis sequence did not deliver the expected product, and the starting material remained unaltered (Scheme 5).

## Conclusion

We demonstrated that the Grignard reaction in combination with the RCM reaction provides a useful strategy for the synthesis of novel and intricate molecular frameworks such as **22**, which is suitable for studying stereoelectronic effects [43]. The strategy shown here is an atom economical process. The synthetic sequence opens up a new route to complex caged systems. At the same time non-participation of **24** in the RRM reaction reveals that systems which contain a sterically hindered cyclohexene ring are not suitable candidates for the tandem metathesis sequence.

## Experimental

### General

Reactions involving organometallic species were carried out under nitrogen by using oven-dried glassware and syringes. THF and Et_2_O were distilled from sodium/benzophenone under nitrogen immediately prior to use. Dichloromethane was distilled over P_2_O_5_. TLC was performed by using (10 × 5 cm) glass plates coated with Acme’s silica gel GF254 (containing 13% calcium sulfate as a binder). Flash-column chromatography was performed by using Aceme silica gel (100–200 mesh). Solvents were concentrated at reduced pressure on a Büchi R-114 rotary evaporator. ^1^H NMR (400 MHz) and ^13^C NMR (75.1 MHz) spectra were recorded at rt on a Bruker AX 400 with TMS (δ = 0.0 ppm, ^1^H NMR spectra) and CDCl_3_ (δ = 77.0 ppm, ^13^C NMR spectra) as internal standards. IR spectra were recorded on a Nicolet Impact-400 FTIR spectrometer. HRMS were determined on a Micromass Q-ToF spectrometer.

#### Materials

Grubbs’ 1^st^ and 2^nd^ generation and Grubbs–Hoveyda catalysts were purchased from Aldrich, Milwaukee (USA). Compounds **18**, **19**, **20**, and **21** were prepared by procedures similar to those described in [28] for analogous compounds.

#### Preparation of 2,5-diallyl-1,4-benzoquinone (**18**)

To a solution of 2,5-diallyl-1,4-hydroquinone (**17**, 1.8 g, 9.45 mmol) in acetone (50 mL) was added MnO_2_ in excess (8 equiv) at rt. After completion of the reaction (TLC monitoring, 15 h), the reaction mixture was filtered off by using a pad of celite. The filtrate was evaporated to dryness and the residue was purified by distillation under reduced pressure at 106–107 °C (1 mmHg) to give **18** as a dark yellow oil (1.2 g, 67%). Bp: 106–107 °C at 1.0 mmHg (Lit. Bp: 105 °C at 1.0 mmHg) [16].

#### Preparation of 2,5-diallyltricyclo[6.2.1.0^2,7^]undeca-4,9-diene-3,6-dione (**19**)

To a cooled solution (at 0 °C) of 2,5-diallyl-1,4-benzoquinone (**18**, 1.17 g, 6.21 mmol) in methanol (25 mL) was added freshly prepared 1,3-cyclopentadiene (0.48 mL, 6.24 mmol) in a dropwise manner. After completion of the reaction (TLC monitoring, 11 h), the solvent was evaporated under reduced pressure, and the residue was purified by silica-gel column chromatography (3% ethyl acetate/petroleum ether) to give **19** (1.13 g, 71.5%) as a thick yellow liquid. IR (Neat) *ν*_max_: 1337, 1666, 2991 cm^−1^; ^1^H NMR (400 MHz, CDCl_3_) δ 1.4 (d, *J* = 8.8 Hz, 1H), 1.6 (d, *J* = 9.2 Hz, 1H), 2.16 (dd, *J_1_* = 8.8 Hz, *J*_2_ = 8.2 Hz, 1H), 2.82 (dd, *J*_1_ = *J*_2_ = 6.7 Hz, 1H), 2.90–3.10 (m, 4H), 3.3 (s, 1H), 4.42–5.12 (m, 4H), 5.92–5.94 (m, 1H), 5.63–5.73 (m, 1H), 5.93 (dd, *J*_1_ = 2.0 Hz, *J*_2_ = 3.0 Hz, 1H), 6.05 (dd, *J*_1_ = 2.0 Hz, *J*_2_ = 3.0 Hz, 1H), 6.3 (s, 1H) ppm; ^13^C NMR (100.6 MHz, CDCl_3_) δ 32.8, 44.9, 46.4, 49.3, 53.1, 54.4, 57.4, 118.2, 132.8, 133.1, 134.9, 137.4, 139.55, 139.58, 152.3, 198.8, 202.0 ppm; HRMS (Q-ToF ES+) *m/z*: calcd for C_17_H_18_O_2_K, 293.0944; found, 293.1086 [M + K]^+^.

#### Preparation of 1,9-diallylpentacyclo[5.4.0.0^2,6^.0^3,10^.0^5,9^]undeca-8,11-dione (**20**)

Tricyclic dione **19** (125 mg, 0.49 mmol) was dissolved in dry ethyl acetate (500 mL) and irradiated in a Pyrex immersion well by a 125 W lamp (homemade) for 1.5 h under nitrogen at rt. After completion of the reaction (TLC monitoring), the solvent was evaporated under reduced pressure and the residue was purified by silica gel column chromatography (5% ethyl acetate/petroleum ether) to give **20** (80 mg, 80%) as a thick pale yellow liquid. IR (KBr) *ν*_max_: 3073, 2974, 1752, 1636, 924 cm^−1^; ^1^H NMR (400 MHz, CDCl_3_) δ 1.82 (d, *J* = 11.4 Hz, 1H), 2.12 (d, *J* = 11.4 Hz, 1H) 2.15–2.4 (m, 6H), 2.55 (dd, *J*_1_ = *J*_2_ = 1.0 Hz, 1H), 2.7 (dd, *J*_1_ = *J*_2_ = 1.0 Hz, 1H), 2.8–3.1 (m, 2H), 5.0–5.2 (m, 4H), 5.6–5.9 (m, 2H) ppm; ^13^C NMR (100.6 MHz, CDCl_3_) δ 33.7, 35.3, 35.6, 39.7, 42.9, 44.0, 47.3, 47.8, 51.5, 60.2, 61.6, 118.5, 118.7, 132.9, 133.7, 212.5, 213.6 ppm; HRMS (Q-ToF ES+) *m/z*: calcd for C_17_H_19_O_2_Na, 277.1204; found, 277.1210 [M + Na]^+^.

#### Preparation of pentacyclic tetraallyldiol **21**

To a freshly prepared solution of allylmagnesium bromide in ether was added an ethereal solution of pentacyclic dione **20** (300 mg, 1.18 mmol) in a dropwise manner over a period of 10–15 min at rt under nitrogen. After completion of the reaction (TLC monitoring, 10 h), the reaction was quenched with a saturated aqueous NH_4_Cl solution at 0 °C. Then, the aqueous layer was extracted by ethyl acetate (3 × 25 mL). The combined organic layer was washed with brine, and dried over anhydrous Na_2_SO_4_. After the removal of the solvent under reduced pressure, the residue was purified by silica gel column chromatography (4% ethyl acetate/petroleum ether) to give **21** (280 mg, 70%) as a white solid. Mp: 158–160 °C; IR (KBr) *ν*_max_: 3309, 3055, 2977, 1265, 743, 705 cm^−1^; ^1^H NMR (400 MHz, CDCl_3_) δ 1.3 (1/2 *AB*q, *J*_1_ = 10.9 Hz, *J*_2_ = 11.0 Hz, 2H), 1.96–2.19 (m, 6H), 2.23–2.46 (m, 8H) 5.08–5.10 (m, 8H), 5.9–6.1 (m, 4H) ppm; ^13^C NMR (100.6 MHz, CDCl_3_) δ 33.1, 35.2, 36.6, 38.1, 41.1, 42.2, 43.1, 44.2, 45.2, 49.0, 49.5, 55.5, 55.7, 78.9, 80.5, 116.71, 116.72, 117.4, 118.3, 134.1, 134.7, 136.1, 136.3 ppm; HRMS (Q-ToF ES+) *m/z*: calcd for C_23_H_30_O_2_Na, 361.2144; found, 361.2146 [M + Na]^+^.

#### Preparation of heptacyclic diol **22**

To a solution of **21** (25 mg, 0.074 mmol) was added Grubbs’ 2^nd^ generation catalyst (4 mg, 6 mol %) under argon at rt. After completion of the reaction (TLC monitoring, 8 h), the solvent was evaporated and the resulting residue was purified by silica gel column chromatography (25% ethyl acetate/petroleum ether) to give **22** (15 mg, 72%) as a white crystalline solid. Mp: 206–207 °C; IR (KBr) *ν*_max_: 3691, 3054, 2987, 2305, 1422, 1266 cm^−1^; ^1^H NMR (400 MHz, CDCl_3_) δ 1.38 (1/2 *AB* q, *J*_1_ = *J*_2_ = 11.0 Hz, 2H), 1.79 (q, *J* = 1.8 Hz, 1H), 1.99–2.56 (m, 13H), 5.54–5.72 (m, 4H) ppm; ^13^C NMR (100.6 MHz, CDCl_3_) δ 31.0, 31.4, 32.4, 36.2, 37.1, 37.7, 44.2, 44.5, 45.1, 47.3, 49.6, 51.4, 58.6, 74.7, 75.0, 123.1, 124.1, 124.4, 126.3 ppm; HRMS (Q-ToF ES+) *m/z*: calcd for C_19_H_22_O_2_Na, 305.1517; found, 305.1523 [M + Na]^+^.

#### Preparation of hexacyclic diallyldiol **24**

To a freshly prepared solution of allylmagnesium bromide (6 equiv) in ether was added the ethereal solution of hexacyclic dione **23** (200 mg, 0.88 mmol) in a dropwise manner over a period of 10–15 min under nitrogen at rt. After completion of the reaction (TLC monitoring, 8 h), the reaction mixture was quenched with saturated aqueous NH_4_Cl solution at 0 °C. Then, the aqueous layer was extracted by ethyl acetate (3 × 25 mL). The combined organic layer was washed with brine and dried over anhydrous Na_2_SO_4_. After removal of the solvent under reduced pressure, the resulting residue was purified by silica-gel column chromatography (4% ethyl acetate/petroleum ether) to give **24** (232 mg, 82%) as a white crystalline solid. Mp: 177–178 °C; IR (KBr) *ν*_max_: 3339, 3054, 2985, 1422, 1265 cm^−1^; ^1^H NMR (400 MHz, CDCl_3_) δ 1.38 (1/2 *ABq*, *J*_1_ = *J*_2_ = 10.3 Hz, 2H), 1.9–2.0 (m, 6H), 2.13–2.25 (m, 6H), 2.32 (d, *J* = 1.6 Hz, 2H), 5.10–5.17 (m, 4H), 5.91–6.02 (m, 4H) ppm; ^13^C NMR (100.6 MHz, CDCl_3_) δ 26.2, 34.7, 42.1, 43.4, 43.7, 48.8, 51.4, 78.3, 117.9, 128.2, 134.0 ppm; HRMS (Q-ToF ES+) *m/z*: calcd for C_20_H_27_O_2_, 311.2011; found, 311.2012 [M + H]^+^.

#### Preparation of hexacyclic diallyldiol **26**

To a freshly prepared solution of allylmagnesium bromide (6 equiv) in ether was added the ethereal solution of hexacyclic dione **25** (250 mg, 1.12 mmol) in a dropwise manner over a period of 10–15 min under nitrogen at rt. After completion of the reaction (TLC monitoring, 8 h), the reaction mixture was quenched with saturated aqueous NH_4_Cl solution at 0 °C. Then, the aqueous layer was extracted with ethyl acetate (3 × 25 mL). The combined organic layer was washed with brine and collected over anhydrous Na_2_SO_4_. After removal of the solvent under reduced pressure, the resulting residue was purified by silica-gel column chromatography (4% ethyl acetate/petroleum ether) to give **26** (292 mg, 85%) as a white crystalline solid. Mp: 191–192 °C; IR (KBr) *ν*_max_: 3339, 3055, 2961, 1439, 1265 cm^−1^; ^1^H NMR (400 MHz, CDCl_3_) δ 0.90 (d, *J* = 10.8 Hz, 1H), 1.40 (d, *J* = 10.8 Hz, 1H), 2.02–2.07 (m, 2H), 2.26 (s, 2H), 2.34–2.39 (m, 4H), 2.73 (s, 2H), 4.84 (brs, 2H), 5.10–5.15 (m, 4H), 5.56 (dd, *J*_1_ = 10.7 Hz, *J*_2_ = 2.7 Hz, 2H), 5.89–6.01 (m, 4H) ppm; ^13^C NMR (100.6 MHz, CDCl_3_) δ 31.7, 42.1, 44.0, 48.6, 51.3, 54.1, 78.8, 118.2, 124.3, 124.4, 133.7 ppm; HRMS (Q-ToF ES+) *m/z*: calcd for C_21_H_25_O_2_, 309.1855; found, 309.1862 [M + H]^+^.

## Supporting Information

File 1 Copies of ^1^H, ^13^C NMR and HRMS spectra for all new compounds.
