# Supplementary material for: Design and synthesis of novel bis-annulated caged polycycles via ring-closing metathesis: pushpakenediol
Source: Beilstein J Org Chem. 2014 Nov 13;10:2664–70. doi: 10.3762/bjoc.10.280 (PMC4273234; doi:10.3762/bjoc.10.280)
Supplement: File 1 — Copies of 1H, 13C NMR and HRMS spectra for all new compounds. [file Beilstein_J_Org_Chem-10-2664-s001.pdf]

# Supporting Information

for

# Design and synthesis of novel bis-annulated caged polycycles via ring-closing metathesis: pushpakenediol

Sambasivarao Kotha* and Mirtunjay Kumar Dipak

Address: Department of Chemistry, Indian Institute of Technology-Bombay, Powai, India, Fax: 022-2572 7152

Email: Sambasivarao Kotha - srk@chem.iitb.ac.in

*Corresponding author

## Copies of $^{1}H$, $^{13}C$ NMR and HRMS spectra for all new compounds

## Table of contents

1. $^{1}H$ and $^{13}C$ NMR spectra of compound **19** S2
2. $^{1}H$ and $^{13}C$ NMR spectra of compound **20** S3
3. $^{1}H$ and $^{13}C$ NMR spectra of compound **21** S4
4. $^{1}H$ and $^{13}C$ NMR spectra of compound **22** S5
5. $^{1}H$ and $^{13}C$ NMR spectra of compound **24** S6
6. $^{1}H$ and $^{13}C$ NMR spectra of compound **26** S7

## 1. $^{1}H$ and $^{13}C$ NMR spectra of compound 19

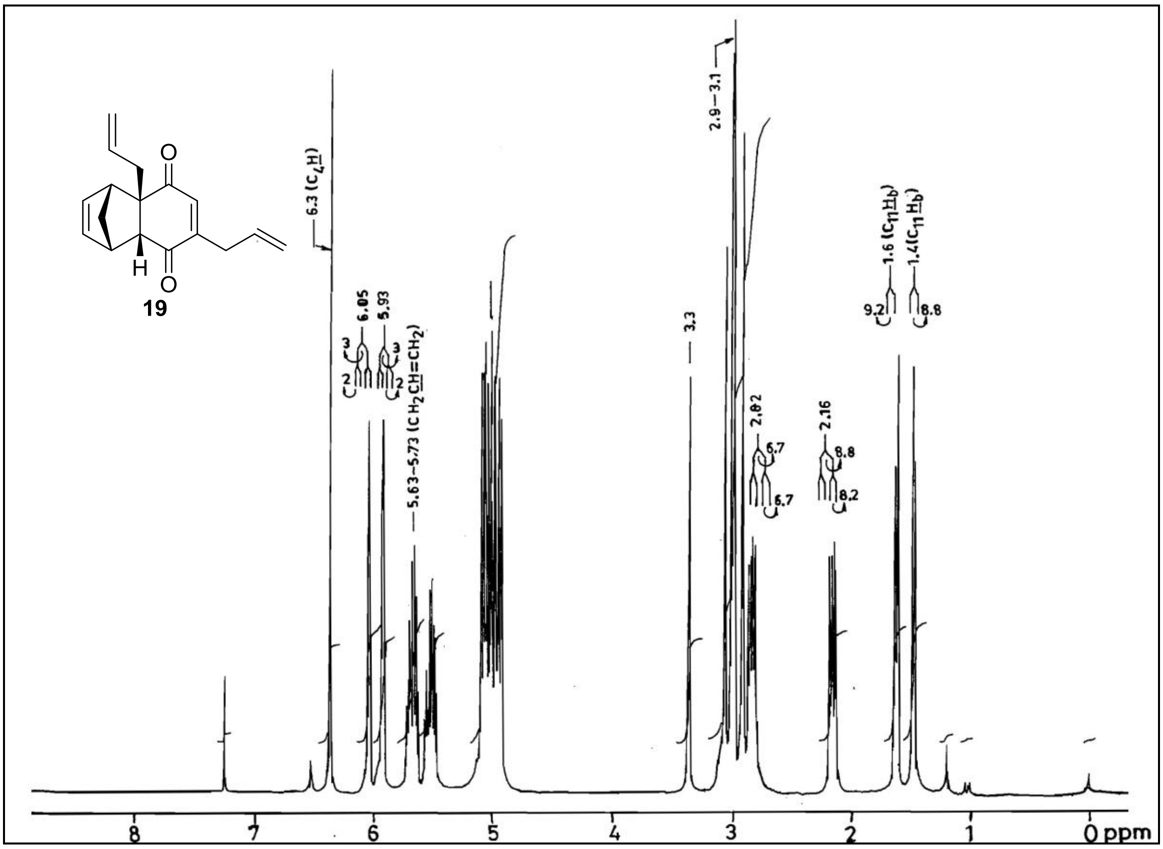


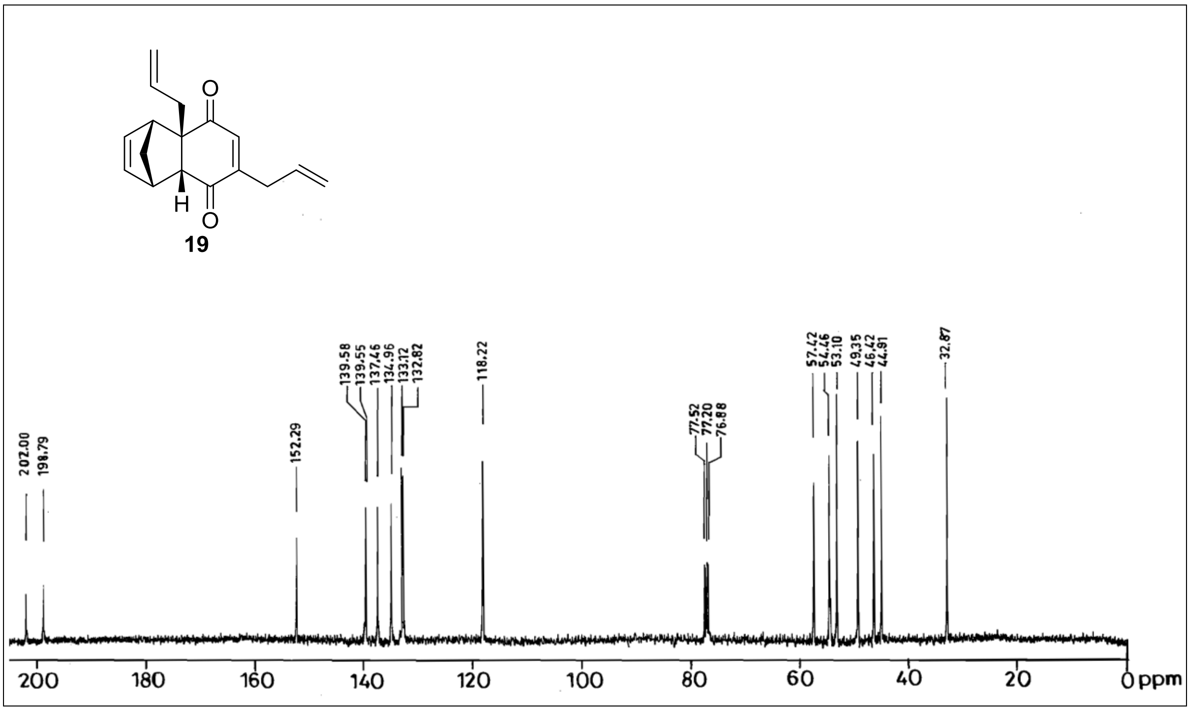

## 2. $^{1}H$ and $^{13}C$ NMR spectra of compound 20

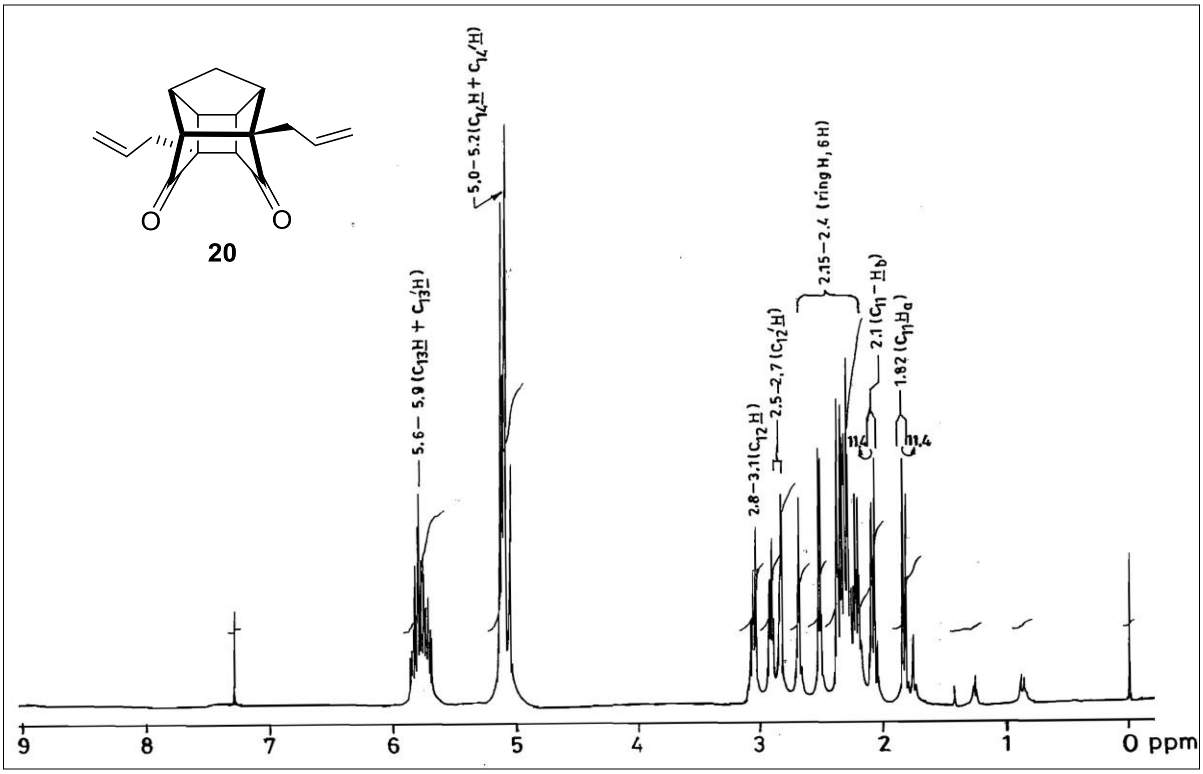


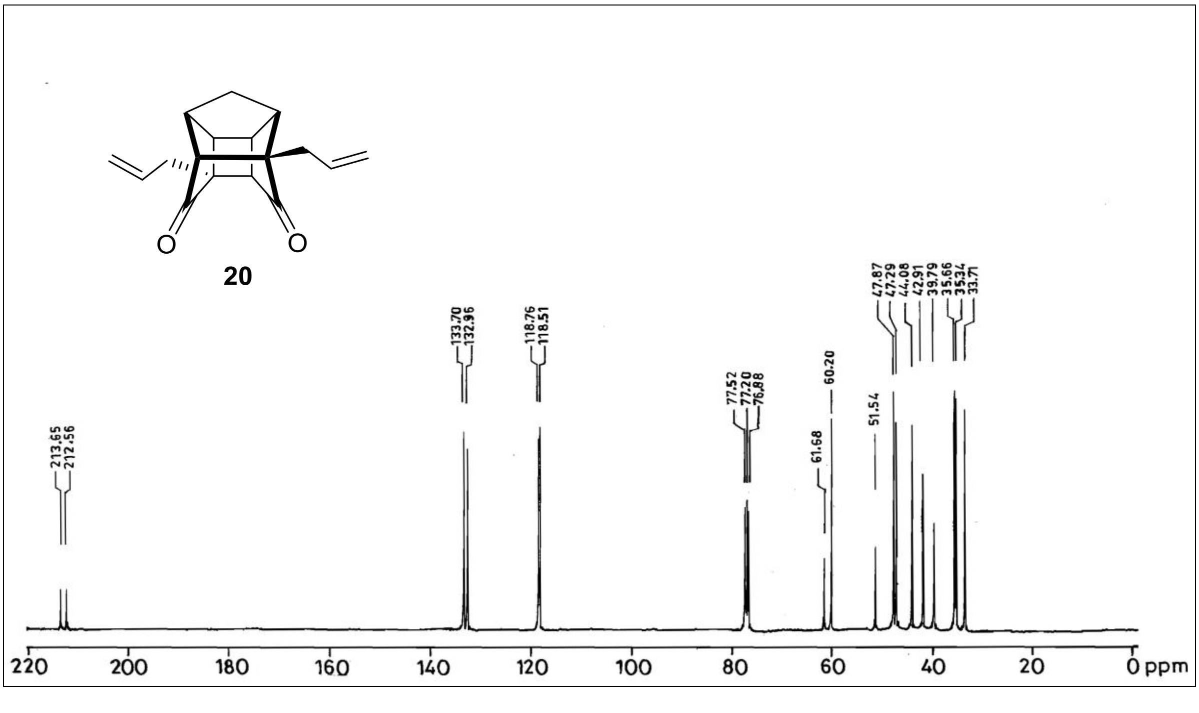

### 3. $^{1}$H and $^{13}$C NMR spectra of compound 21

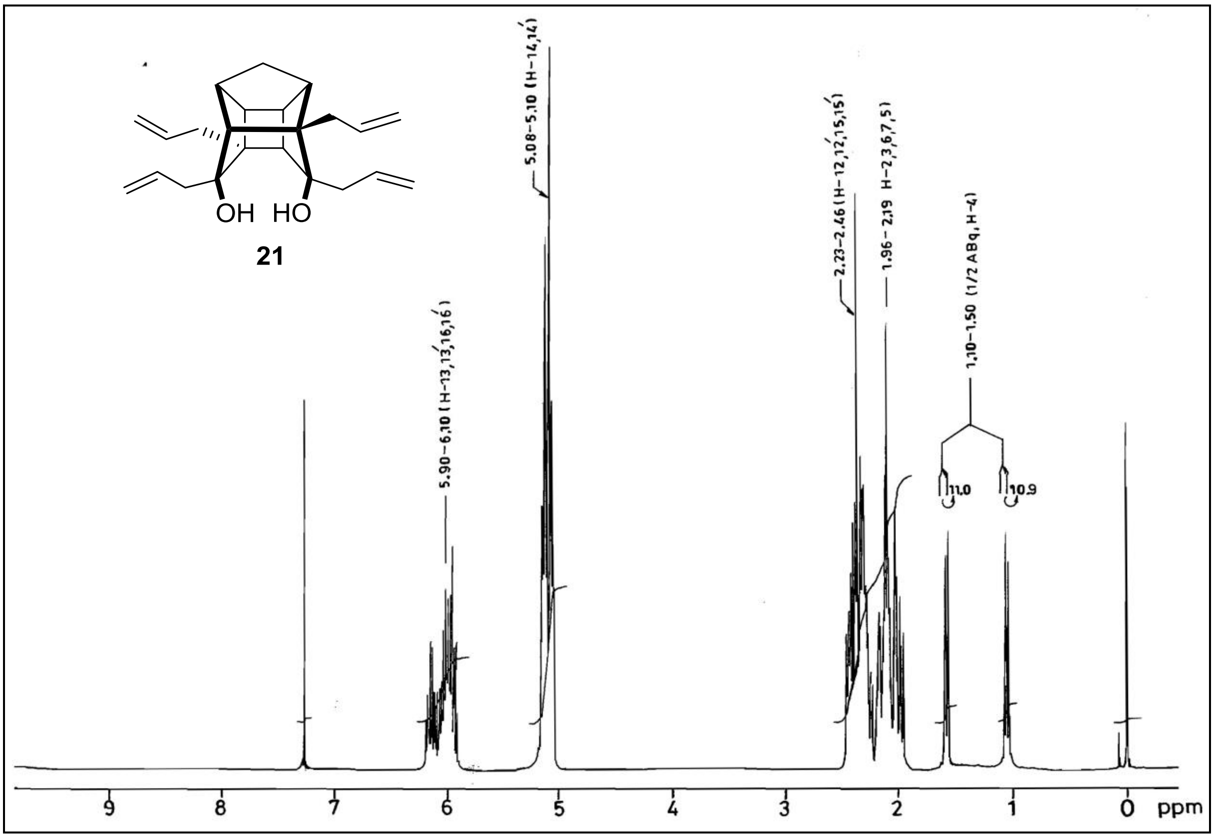


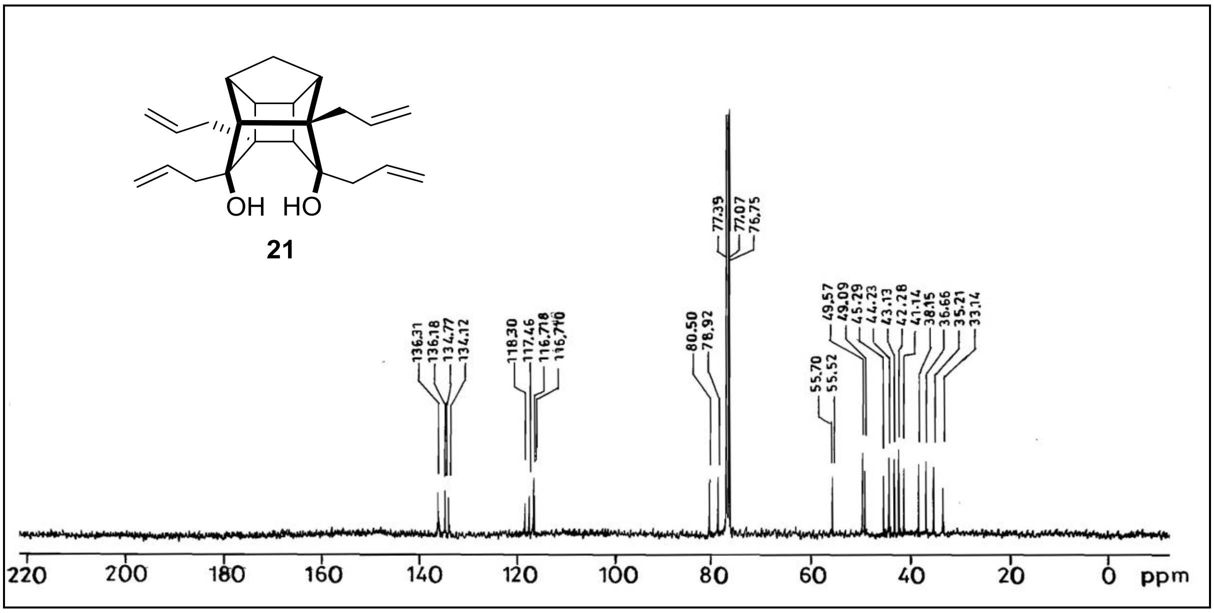

# 4. $^{1}$H and $^{13}$C NMR spectra of compound 22

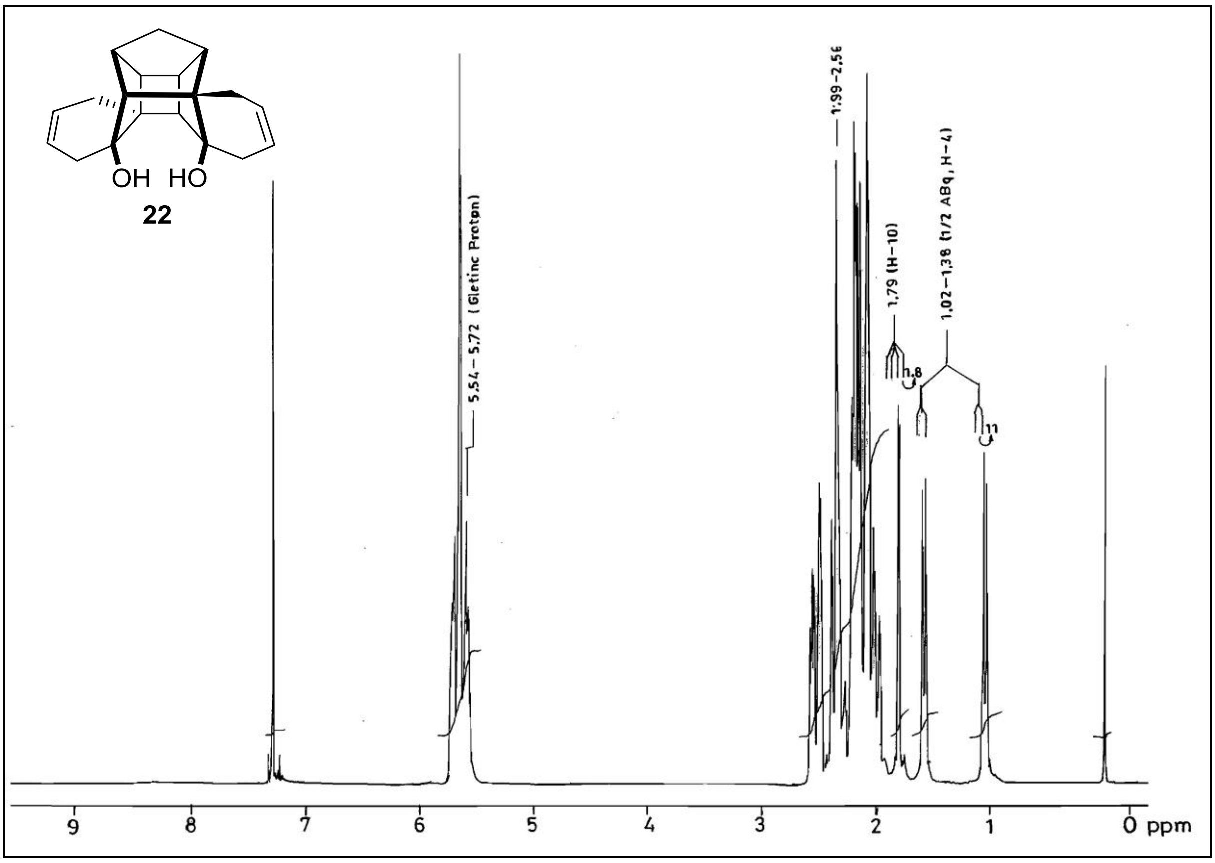


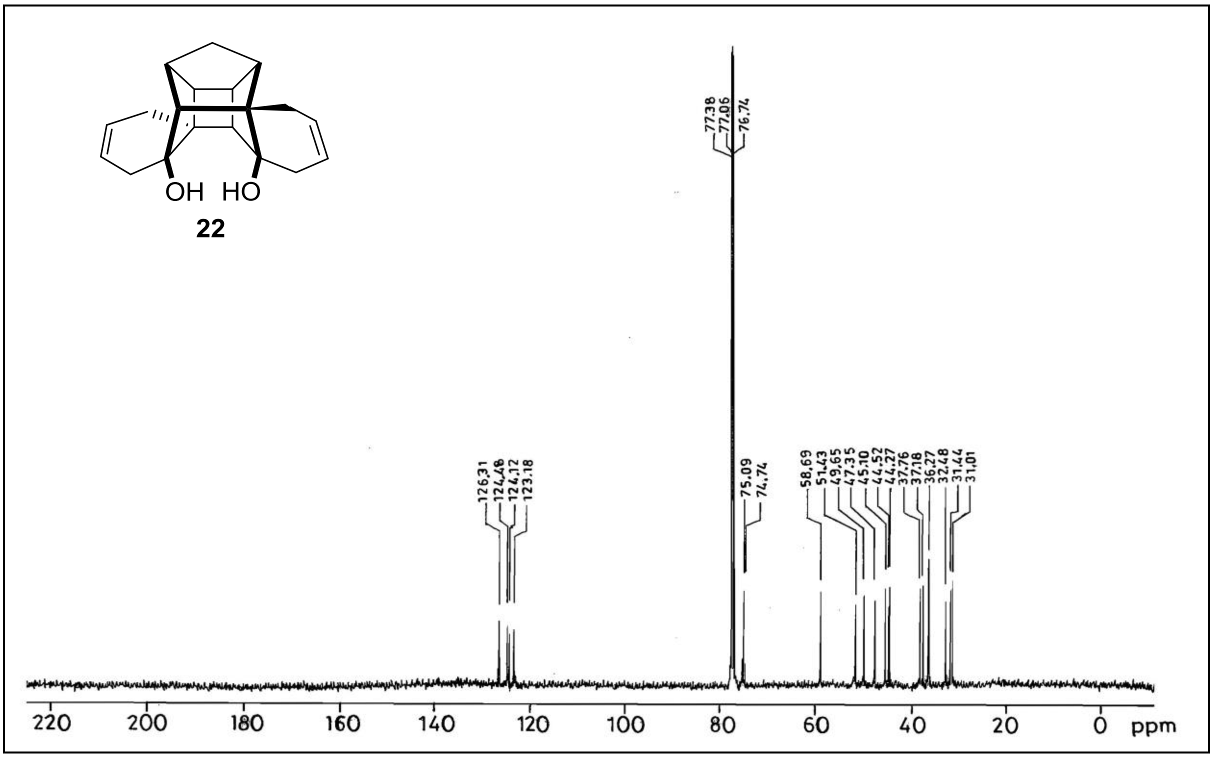

## 5. $^{1}$H and $^{13}$C NMR spectra of compound 24

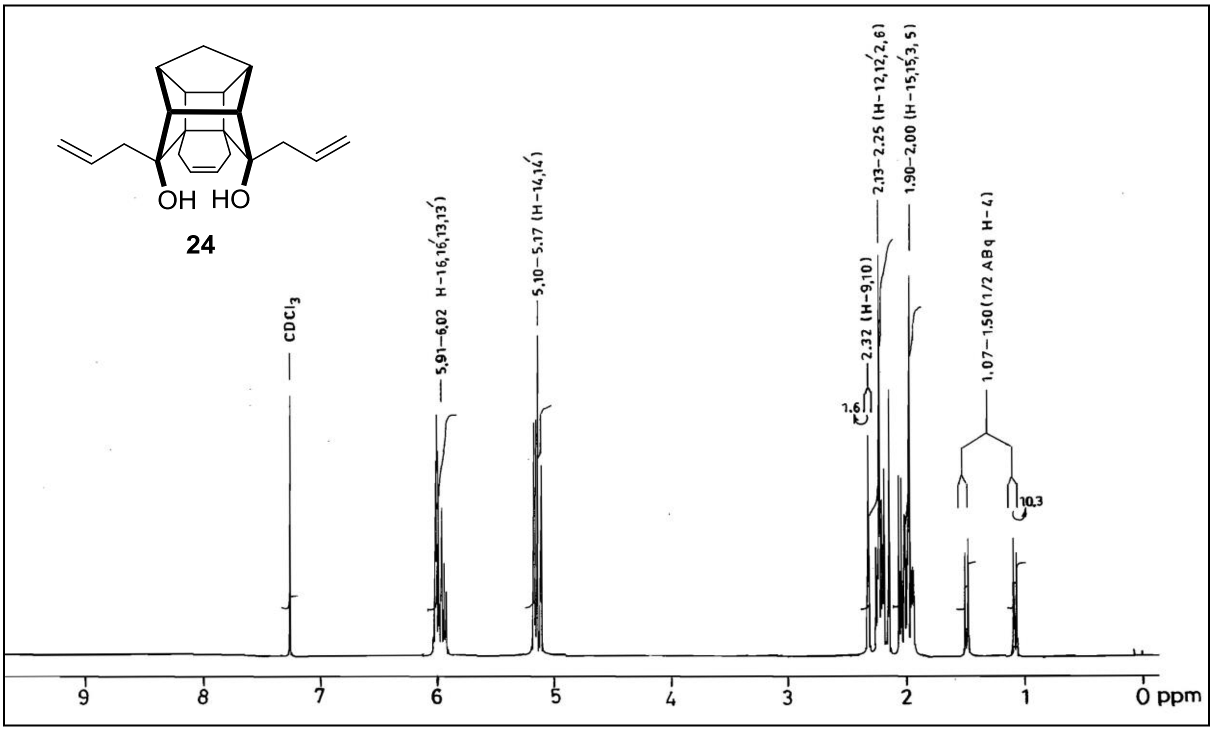


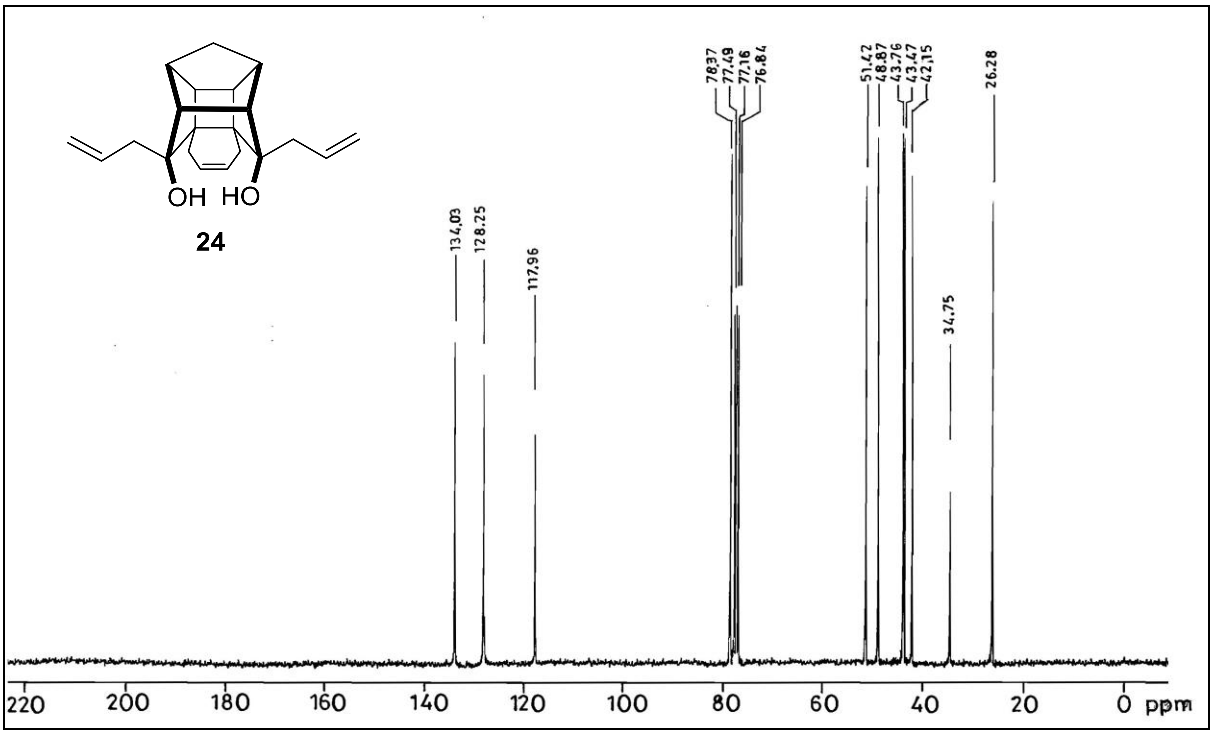

# 6. $^1$H and $^{13}$C NMR spectra of compound 26

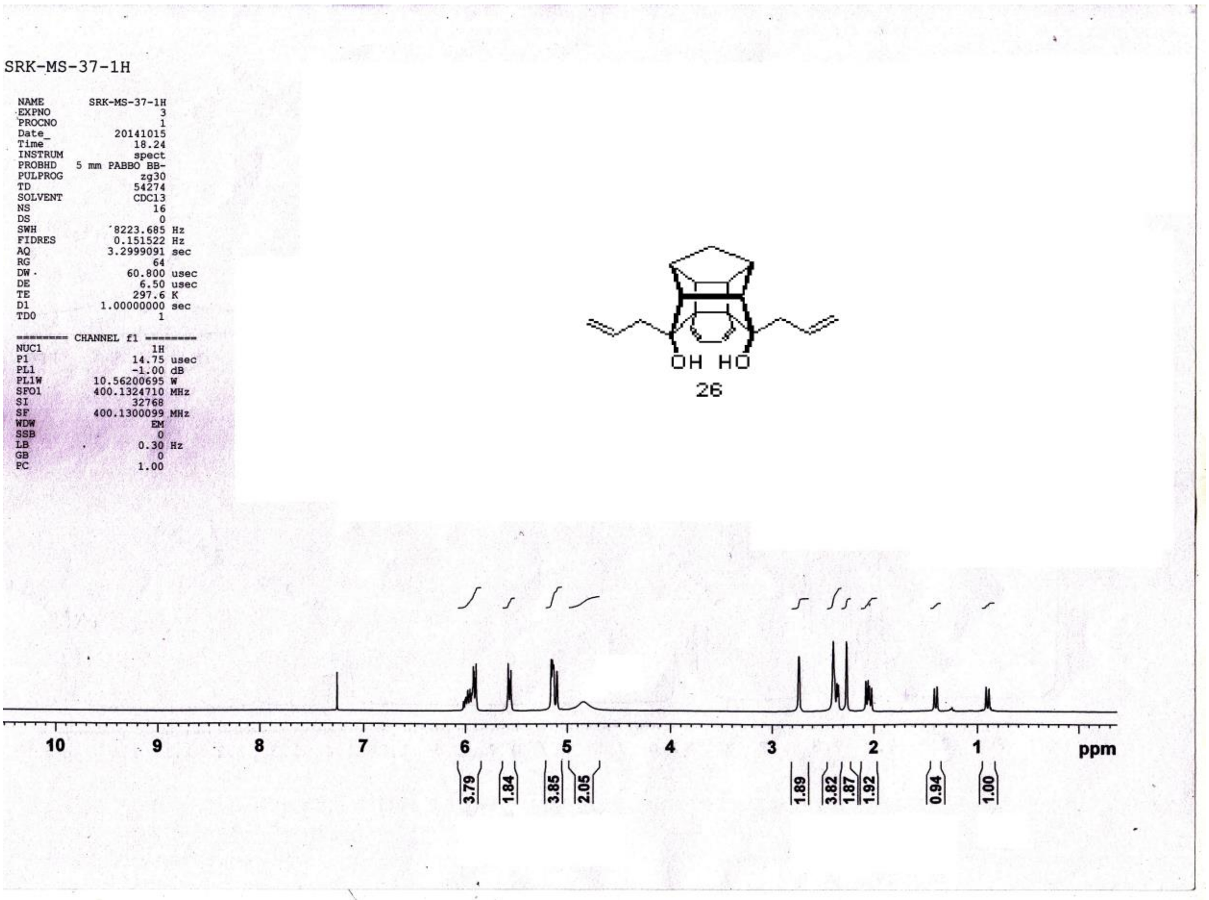

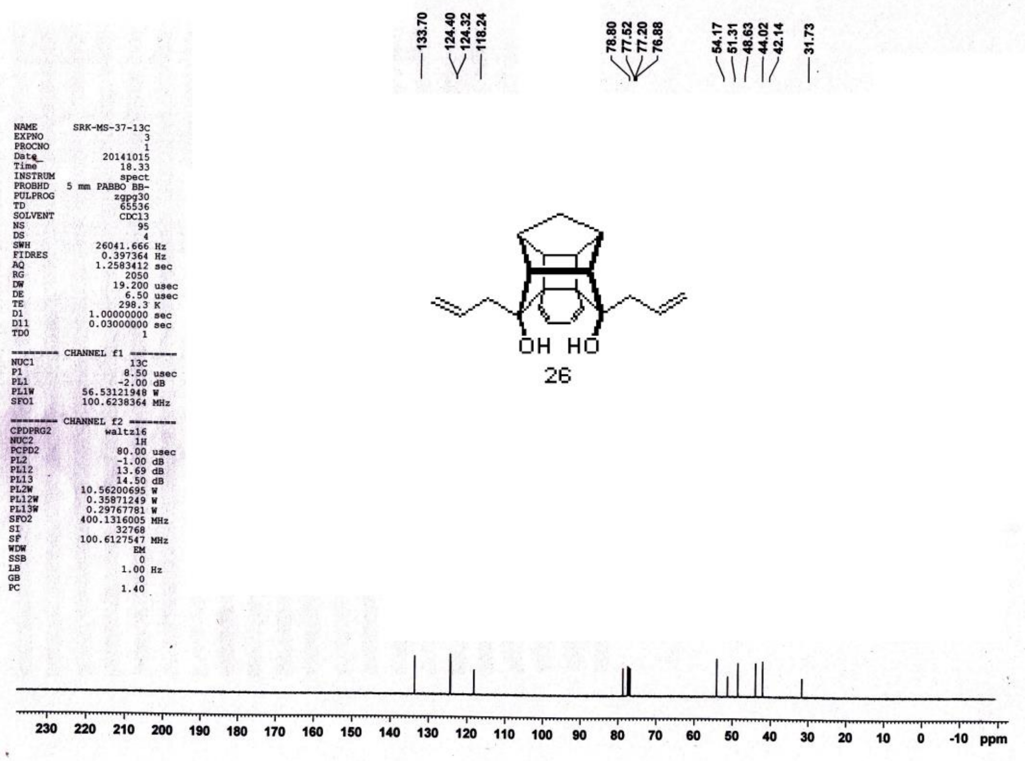
133.70
124.40
124.32
118.24
78.80
77.52
77.20
76.88
54.17
51.31
48.63
44.02
42.14
31.73
NAME SRK-MS-37-13C
EXPNO 3
PROCNO 1
Date 20141015
Time 18.33
INSTRUM spect
PROBHD 5 mm PABBO BB-
PULPROG zgpg30
TD 65536
SOLVENT CDCl3
NS 95
DS 4
SWH 26041.666 Hz
FIDRES 0.397364 Hz
AQ 1.2583412 sec
RG 2050
DW 19.200 usec
DE 6.50 usec
TE 298.3 K
D1 1.00000000 sec
D11 0.03000000 sec
TD0 1
======== CHANNEL f1 ========
NUC1 13C
P1 8.50 usec
PL1 -2.00 dB
PL1W 56.53121948 W
SFO1 100.6238364 MHz
======== CHANNEL f2 ========
CPDPRG2 waltz16
NUC2 1H
PCPD2 80.00 usec
PL2 -1.00 dB
PL12 13.69 dB
PL13 14.50 dB
PL2W 10.56200695 W
PL12W 0.35871249 W
PL13W 0.29767781 W
SFO2 400.1316005 MHz
SI 32768
SF 100.6127547 MHz
WDW EM
SSB 0
LB 1.00 Hz
GB 0
PC 1.40
OH HO
26
230 220 210 200 190 180 170 160 150 140 130 120 110 100 90 80 70 60 50 40 30 20 10 0 -10 ppm
